# Molecular methods in cancer diagnostics: a short review

**DOI:** 10.1080/07853890.2024.2353893

**Published:** 2024-05-16

**Authors:** Tanushree Budhbaware, Jaishriram Rathored, Sandesh Shende

**Affiliations:** Department of ‘School of Allied Health Sciences’, Central Research Laboratory (CRL) and Molecular Diagnostics, Datta Meghe Institute of Higher Education and Research, Sawangi (Meghe), Wardha, India

**Keywords:** Molecular techniques, cancer genetics, cancer diagnosis, polymerase chain reaction, next generation sequencing

## Abstract

**Background:**

One of the ailments with the greatest fatality rates in the 21st century is cancer. Globally, molecular methods are widely employed to treat cancer-related disorders, and the body of research on this subject is growing yearly. A thorough and critical summary of the data supporting molecular methods for illnesses linked to cancer is required.

**Objective:**

In order to guide clinical practice and future research, it is important to examine and summarize the systematic reviews (SRs) that evaluate the efficacy and safety of molecular methods for disorders associated to cancer.

**Methods:**

We developed a comprehensive search strategy to find relevant articles from electronic databases like PubMed, Google Scholar, Web of Science (WoS), or Scopus. We looked through the literature and determined which diagnostic methods in cancer genetics were particularly reliable. We used phrases like ‘cancer genetics’, genetic susceptibility, Hereditary cancer, cancer risk assessment, ‘cancer diagnostic tools’, cancer screening’, biomarkers, and molecular diagnostics, reviews and meta-analyses evaluating the efficacy and safety of molecular therapies for cancer-related disorders. Research that only consider treatment modalities that don’t necessitate genetic or molecular diagnostics fall under the exclusion criteria.

**Results:**

The results of this comprehensive review clearly demonstrate the transformative impact of molecular methods in the realm of cancer genetics.

This review underscores how these technologies have empowered researchers and clinicians to identify and understand key genetic alterations that drive malignancy, ranging from point mutations to structural variations. Such insights are instrumental in pinpointing critical oncogenic drivers and potential therapeutic targets, thus opening the door for methods in precision medicine that can significantly improve patient outcomes.

**Limitation:**

The search does not specify a timeframe for publication inclusion, it may have missed recent advancements or changes in the field’s landscape of molecular methods for cancer. As a result, it may not have included the most recent developments in the field.

**Conclusion:**

After conducting an in-depth study on the molecular methods in cancer genetics, it is evident that these cutting-edge technologies have revolutionized the field of oncology, providing researchers and clinicians with powerful tools to unravel the complexities of cancer at the genetic level. The integration of molecular methods techniques has not only enhanced our understanding of cancer etiology, progression, and treatment response but has also opened new avenues for personalized medicine and targeted therapies, leading to improved patient outcomes.

## Introduction

Whenever a cell’s normal cycle regulatory system deteriorates, a medical disorder known as carcinoma develops, which is represented by unusual cell growth and eventual death [1]. Human health and safety depend on early cancer detection and precise treatment [2]. The need for innovative technologies has evolved in response to this tremendous development. For instance, Polymerase Chain reaction (PCR,) Next generation sequencing (NGS,) CT, MRI, Gel electrophoresis, PET, and X-ray might produce precise visualizations underneath the structures of the body’s internal components to understand any of these anomalies, and it is estimated that there are about 100 unique kinds of cancer [3]. These include abnormalities of the blood, organs, bones, etc.; however, it is still unclear what causes cancer and how it affects healthy creatures. The categorization of illnesses has improved with a deeper understanding of their origins and symptoms, such as local tumors and systemic variations [3]. The course of treatment for related malignancies should be carefully modified depending on the specific features of each type of carcinoma. The most prevalent traditional cancer treatment options include surgical removal, chemotherapy, radiation, and others [4]. Because the margins of carcinoma tissue are difficult to discern during surgery, it is difficult to completely remove the cancer cells, which might result in tumor recurrence [5]. Chemotherapy and radiation have considerable adverse consequences [3]. Cancer is immediately connected to intestinal flora [6]. The intestinal flora has a footprint on cancer onset and counselling, efficiency, and side effects of cancer immune therapy [7]. The ability of carcinoma cells to respond to fluctuations in oxygen tension is essential for tumor growth [8].

The likelihood of acquiring malignancies among individuals is substantially correlated with folate consumption [9]. Numerous foods, including legumes, cereals, fruit, and green leafy vegetables, contain water-soluble vitamins in the B vitamin family that are essential for halting the progression of human illnesses such as adult cardiovascular disease and malignancies, geriatric dementia and cognitive decline, and prenatal genetic defects [9].

A small percentage of individuals can benefit from present-day treatment with chemotherapy, radiation treatment, and surgery, which are typical cancer-related treatments [10]. This is owed to the fact that not all kinds of cancer are brought on by the identical the genome, and that everyone’s tumor is diverse [11]. Owing to the intricacy of their mechanisms, alternative therapy modalities are becoming increasingly significant. It is interesting to note that genome editing has been used to treat a variety of ailments. Clustered regularly interspaced short palindromic repeats (CRISPR/Cas9) have been researched, particularly for the treatment of Non-Small Cell Lung Carcinoma (NSCLC) [12]. The use of CRISPR/Cas9 genetic editing technology for transcriptomic legislation, expression replacement, gene knock-in, and gene knock-out has proven effective [11]. MALAT1 (Metastasis Associated Lung Adenocarcinoma Transcript 1) is a widely recognized Long non-coding RNA (lncRNA) in cancer [13].

In humans, MALAT1 is encoded on chromosome 11q13 [14].

In order to conduct this literature review, we searched the PubMed, Web of Science (WoS) and Scopus original articles as well as review article using the phrases ‘cancer genetics’, genetic susceptibility, Hereditary cancer, cancer risk assessment ‘cancer diagnostic tools’, cancer screening, biomarkers, molecular diagnostics and we looked through the literature and determined which diagnostic methods in cancer genetics were particularly reliable. Meta-analyses and systematic reviews assessing the safety and effectiveness of molecular treatments for conditions related to cancer were also included in the study. Studies that are limited to treatment approaches that do not require genetic or molecular diagnostics will not include in current review and considered as exclusion criteria.

### Molecular techniques

Technological and biological science advancements have led to the creation of molecular targets for cancer monitoring and therapy [15]. The Sensitivity and accuracy for the identification of uncommon DNA copies and pattern variants have significantly increased owing to techniques such as PCR and tailored NGS [16].

### Polymerase chain reaction (PCR)

Digital PCR (dPCR), real-time quantitative PCR (qPCR), and reverse transcriptase PCR (RT-PCR) mass spectrometry-based approaches are the three main subcategories of PCR-based methods [17]. When DNA anomalies are sufficiently consistent, PCR has been effectively used to detect low quantities of tumor cells in haematological malignancies [18].

qPCR research has grown with the invention of gadgets that integrate microarray technology. Amplification and analysis are both included in homogeneous real-time quantitative PCR technology, which requires no sample, radioactivity, or slab jelly processing [15]. The amplification process of PCR and fluorescence monitoring of each cycle (A) compares the quantities of the target in a sample that is both experimental and control. Genomic DNA analysis can be performed to evaluate gene amplification or deletions. Research on mRNA expression after reverse transcription has been conducted. Samples containing more DNA (or cDNA) exhibited fluorescence that increased earlier. The curves’ second derivative maxima (vertical dotted lines) are located as fractional cycle numbers. The difference in fractional cycle numbers between samples is that the PCR efficiency increased with the relative copy number within them. The computation is based on the idea that the PCR efficiency of each sample is the same. Typically, PCR efficiency ranges from 1.7 to 2.0. It is common to assume an efficiency of two as the first approximation. This technique is based on the idea that each sample contains an equal initial concentration of material (cDNA or DNA). (B) Using a test target normalized to a reference target is an additional option. Every sample’s starting material quantity was standardized to a housekeeping or reference gene. Amplified versions of the experimental and control samples were used as test and reference targets. The reference target amplification results normalized any variation in the amount of starting material. The PCR efficiency of each target remained constant across samples, and the reference target was assumed to be invariant in this technique. The ΔΔC technique is named after the assumption of an efficiency of 2 for both goals as the first estimate, as shown in Figure 2 [15].

Gene duplications and deletions can be detected using quantitative real-time PCR [15]. Moreover, minor mutations down to single-base alterations can be found by melting curve analysis performed just after PCR [19]. Real-time PCR is a good choice for cancer marker is real-time PCR. Internal controls may be offered, reagent costs may be reduced, and priceless samples can be preserved compared to single-target analysis [20].

**Figure 1.**
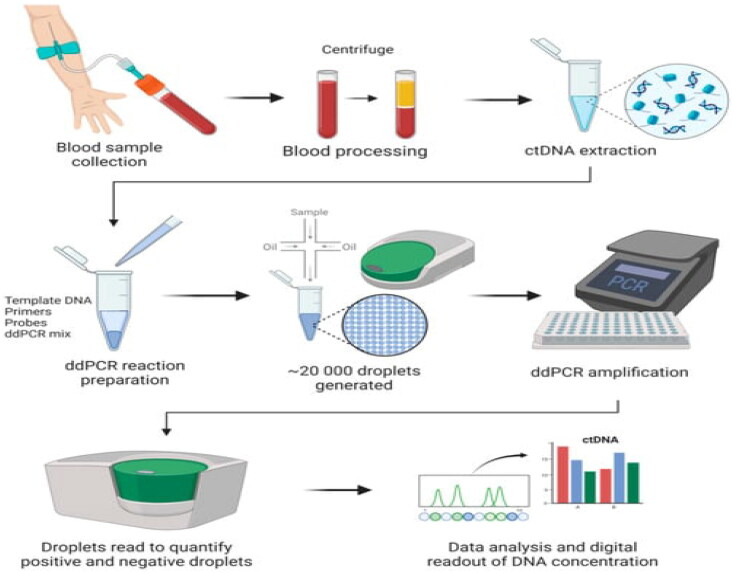
Typical workflow for ddPCR in cancer liquid biopsies (Adapted from Ugur Gezer et al.) [22].

qPCR is frequently used because it is quick and reasonably priced [21]. However, only MAFs larger than 10% can be used to identify mutant alleles [17]. The most recent iteration of PCR is known as ddPCR (droplet digital PCR) work flow were shown in Figure 1. Similar to qPCR, the theory behind d-PCR divides the material into hundreds simultaneous PCR studies to cut down on background chatter, As a result, It is able to identify MAF levels below a value of 0.1% [17]. Concordant downregulation of miR-497 in tumor tissues and plasma has been confirmed by quantitative RT-PCR analysis, implying that plasma miR-497 might be employed as a biomarker for the diagnosis of carcinoma of the nasopharyngeal tract [23]. Patients with melanoma and neuroblastoma may benefit from the identification of a small number of circulating cells using RT-PCR for tissue-specific gene expression [18]. RT-PCR strategy has the capacity to raise Monitoring for breast cancer cells sensitivity to as low as a few cells per million [24]. Experiments with spikes in peripheral volume of blood, 3 ml from healthy, normal individuals at dimensions of MCF-7cells of 0, 1, 10, 102, 103, 104, and 105. The product sizes of cytokeratin protein 19 (KRT19), mammaglobin (MGB), and parathyroid hormone-related peptide (PTHRP) are 186, 159, and 367 bp, respectively. RT-PCR was conducted with a sensitivity of 10 MCF-7 cancer cells per three millilitre of peripheral blood (Figure 3) [25].

**Figure 2.**
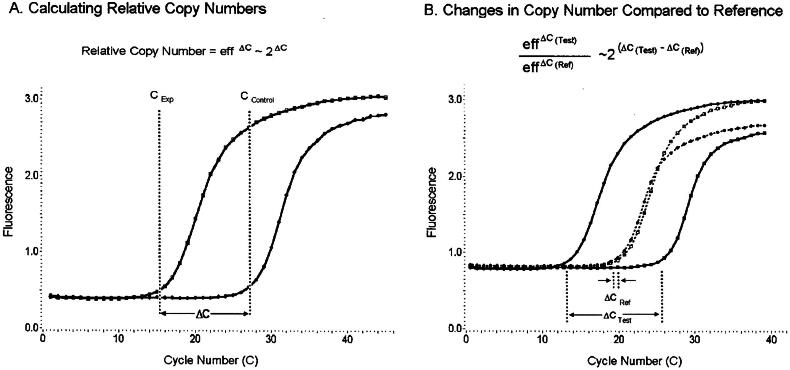
Real-time PCR-based with regard quantifying (Adapted from Bernard PS et al.) [15].

**Figure 3.**
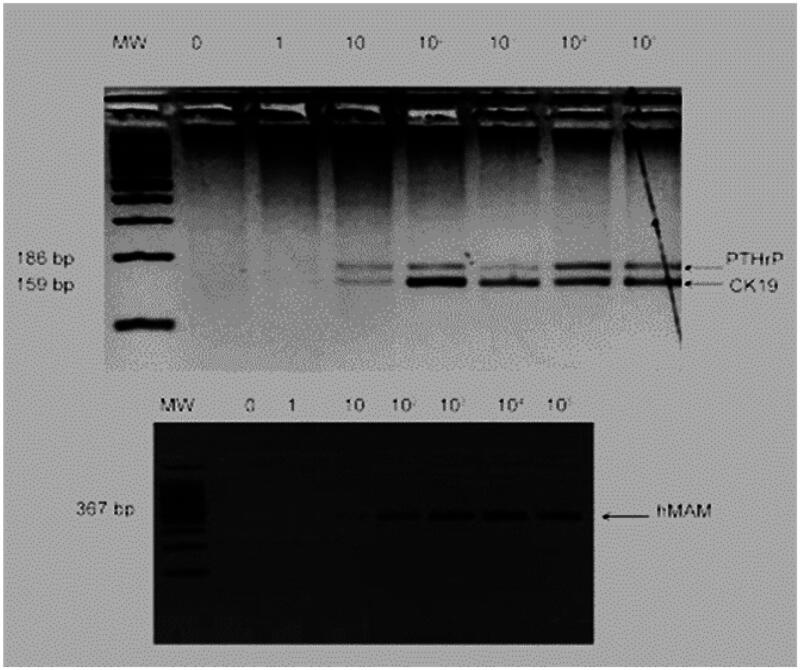
RT-PCR (Adapted from Skondra M et al.) [25].

For patients with mutations or amplifications/deletions, dd-PCR can provide an accurate approach for measuring circulating tumor DNA (ctDNA) target sequences in liquid biopsies that are both fast and inexpensive [16]. ddPCR has a wide range of oncology applications, and breast carcinoma will likely gain significantly from this methodology [26]. In a research of Twenty-nine people with preliminary breast cancer, the effectiveness of ptDNA (plasma Tumor DNA)/ddPCR for identifying PIK3CA mutations was established with 93.3% sensitivity and 100% specificity [27].

Possessing a distinct advantage in multiplex detection, this mass spectrometry-based approach is a modification of the traditional PCR methodology, in addition to qPCR and dPCR [17]. For instance, with a MAF as low as 0.1%, Ultra SEEK can identify mutant sequence mixes [28]. Multiplex PCR was initially used to simultaneously amplify all mixtures [29]. Then, using time-of-flight mass spectrometry *via* matrix-assisted laser desorption/ionization, mutations are detected using the designated chain terminators of the single-base extension [17].

### Maxam-Gilbert sequencing method

**Figure 4.**
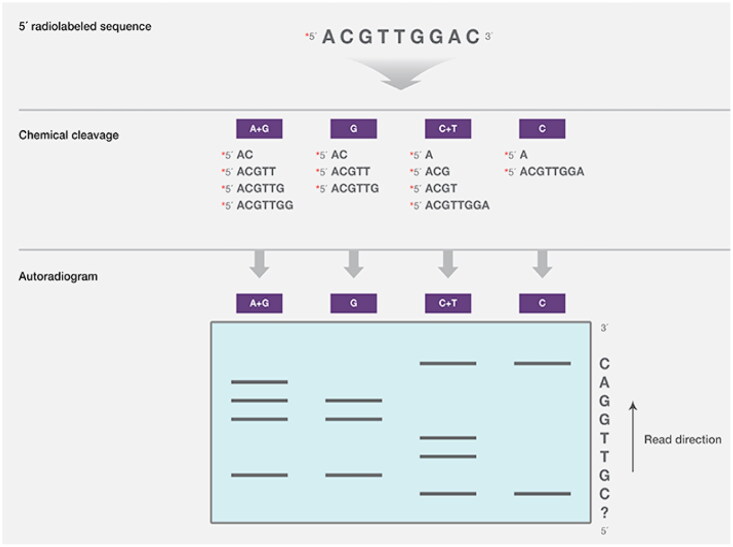
Maxam-Gilbert DNA sequencing method (Adapted from Thermo Fisher Scientific: DNA Sequencing Technologies–History and Overview) [30].

DNA segments with radiolabeled 5′ ends are chemically cleaved at different nucleotides (including G and A, C and T, or C and G) using the Maxam-Gilbert procedure (Figure 4). Next, cleavage fragments were identified by autoradiography and separated by gel electrophoresis [31].

### Sanger sequencing method

**Figure 5.**
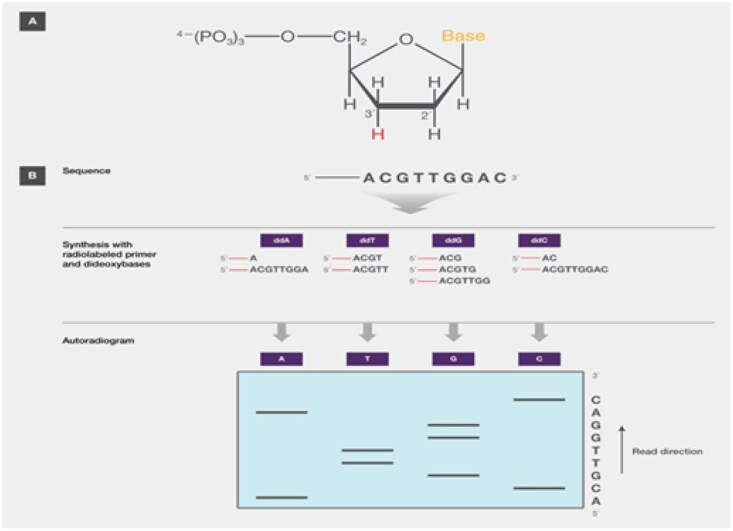
Sanger DNA sequencing method (Adapted from Thermo Fisher Scientific: DNA Sequencing Technologies–History and Overview) [30].

In the Sanger method (Figure 5), radiolabelled oligonucleotides are utilized to stimulate DNA synthesis utilizing 2′,3′-dideoxynucleoside (ddNTP) as the source of modified nucleotide triphosphate. ddNTP, sometimes referred to as dideoxy, breaks the developing chain because, in order to combine with an arriving normal nucleotide to form a phosphodiester bond (deoxy nucleoside triphosphate, or dNTP), it lacks the 3′-hydroxyl group. The reaction mix had a low quantity of ddNTPs; thus, instead of adding conventional dNTPs at random, ddNTPs were added randomly, generating recently synthesized DNA fragments of different lengths. Then, in a process akin to the Maxam-Gilbert approach, electrophoresis on a gel is used to distinguish these small pieces and identify them by autoradiography. The Sanger technique has become the industry standard for sequencing because it is theoretically simpler than the Maxam-Gilbert approach [32]. The most prestigious single-nucleotide variation and minor insertions/deletions are Sanger sequencing [33]. However, it is ineffective for significant rearrangements, invasive additions, and deletions. NGS can completely detect all target gene mutations, including chromosomal abnormalities [34].

### Automated sequencing method

With the arrival of automated DNA sequencing, in which fluorescent dye-labelled nucleotides and capillary electrophoresis-based DNA fragment separation are performed, Sanger sequencing has become increasingly common [35]. One crucial factor in determining the cellular mode of action of a drug is the sequence specificity of the DNA-damaging agent. Many substances that damage DNA are carcinogenic and mutagenic, and thus, are frequently employed as cancer-fighting chemotherapy drugs [36]. The specificity of the DNA sequences of some agents that damage DNA has been ascertained using DNA sequencing technology [36].

### Next generation sequencing method (NGS)

**Figure 6.**
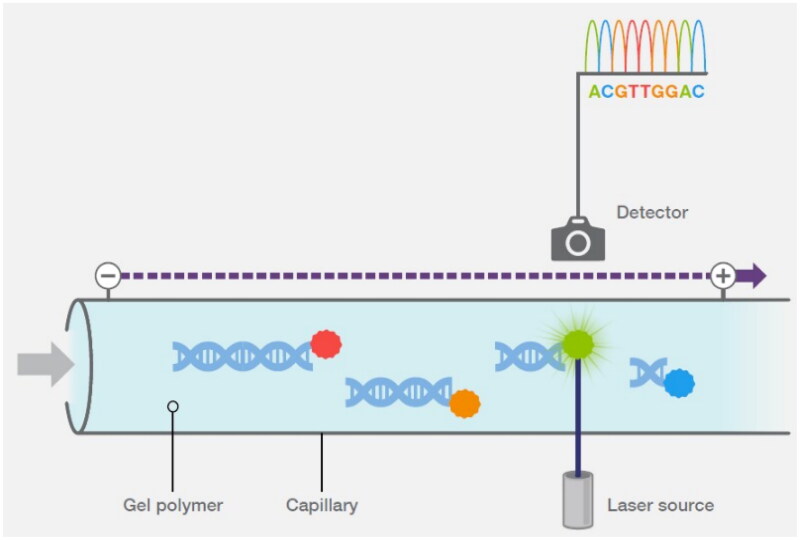
Capillary electrophoresis (Adapted from Thermo Fisher Scientific: DNA Sequencing Technologies–History and Overview) [30].

NGS enables the sequencing of 150–400 base DNA segments in massive parallel. With this approach, as a DNA polymerase creates complementary strands, template DNA fragments are amplified clonally and sequenced in real time, as opposed to being read *via* gel or capillary electrophoresis (Figure 6). Sequencing by synthesis or SBS is another type of sequencing strategy [37].

RNA sequencing, minimal frequency bisulfite sequencing, immunoprecipitation of chromatin sequencing, and whole-exome sequencing of genes constitute a few NGS strategies [11]. For example, the most suitable candidates for aromatase inhibitor treatment can be identified using breast cancer tumor sequencing [38]. Modern oncology mostly uses NGS to identify driver mutations that lead to oncogene addiction and to differentiate between driver and passenger mutations [39]. The tumor was removed using immunotherapy, and somatic cell mutations were detected using NGS [37]. TP53, PIK3CA, and GATA3 also frequently harbor somatic mutations in breast cancer [11]. NGS permits the sequencing of an enormous number of nucleotides in just a brief amount of time and at affordable prices per participant [40].

The NGS platform Ion Torrent was launched by Thermo Fisher Scientific [41]. It permits the detection of copy number variations (CNVs), single-nucleotide polymorphisms (SNPs), indels, and fusions with as little as 1 ng DNA input [17]. The limitations of situations with limited resources must be considered when using NGS in poor nations [42]. Patients with lung cancer and breast cancer will unquestionably benefit from NGS prognostic information, in addition to therapy recommendations [43]. Next-generation genome sequencing general and NGS a priori’ methods: contrast and use (Figure 17) has been adopted in clinical practice for recognizing driver and resistance variants in malignancies such as lung cancer, breast cancer, and cancers of unknown genuine origin [45]. Stakeholders need to work together to integrate this technology into health systems to enhance cancer care, as international standards support NGS [46]. The use of NGS identifies several existing issues in the Colombian healthcare system and offers the opportunity for comprehensive cancer treatment [43].

Recently, the European Society for Medical Oncology (ESMO) published guidelines recommending NGS for patients with advanced cancer [47]. The deployment of diagnostic NGS for NSCLC in Asia aims to provide individuals with access to the most appropriate customized medical care [48].

**Figure 7.**
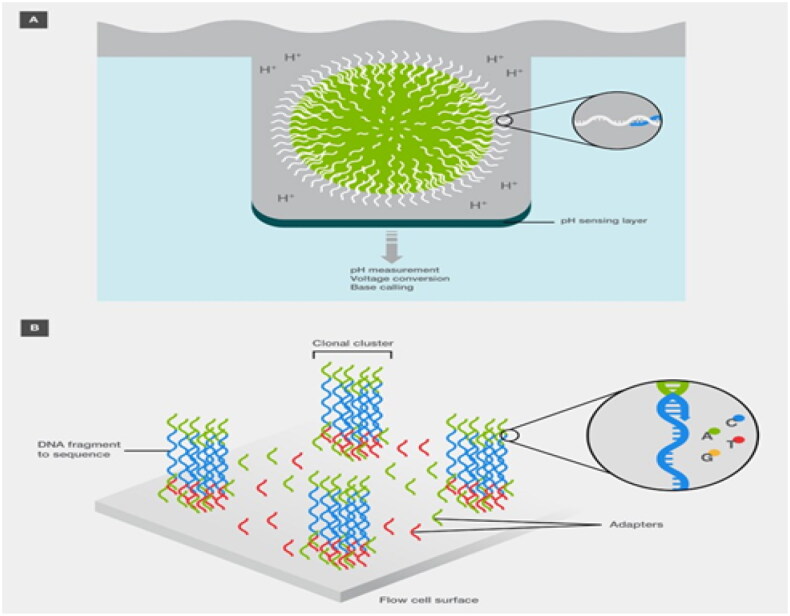
(A) Technology for Ion Torrent sequencing and (B) technology for Illumina sequencing (Adapted from Thermo Fisher Scientific: DNA Sequencing Technologies–History and Overview) [30].

NGS and PCR are diagnostic tools [49]. Nevertheless, NGS is more precise than PCR for detecting genome alterations, whereas real-time PCR techniques were unable to detect one insertion-deletion and seven nonsynonymous single-nucleotide shift variations, and NGS proved capable of doing so [11]. The input and speed of PCR-based approaches are constrained; they are only able to search for known variations and are sensitive and affordable [50]. High-throughput NGS can screen for unidentified modifications [11]. (A) Technology for Ion Torrent sequencing. Sequencing clonally amplified DNA on a bead placed in each well of a semiconductor chip involves tracking changes in the solution pH as each nucleotide combines and releases an H + ion. (B) Technology used for Illumina sequencing. Clonal clusters are formed on a flow cell, and the fluorescence emission of each nucleotide is used to identify the sequence shown in Figure 7 [30].

### Single-molecule real-time technology (SMRT)

**Figure 8.**
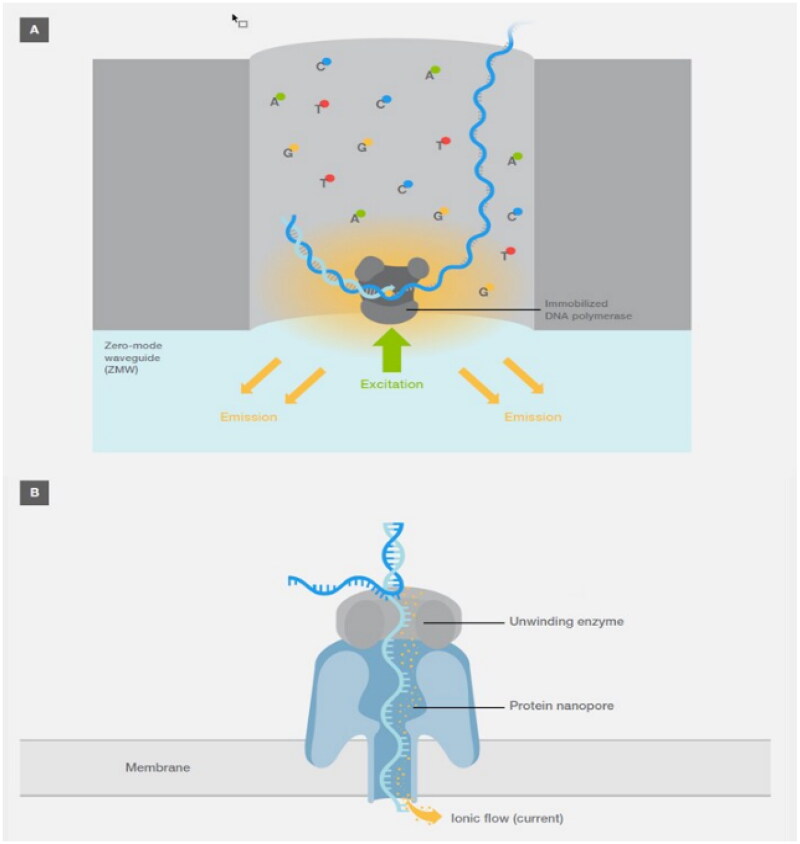
(A) The technology of SMRT sequencing and (B) the technology of nanopore sequencing (Adapted from Thermo Fisher Scientific: DNA Sequencing Technologies–History and Overview) [30].

One base at a time, Pacific Biosciences’ SMRT technology, uses fluorescence to identify when a DNA polymerase extends a molecule. An alternative technique called nanopore sequencing, developed by Oxford Nanopore Technologies, breaks up the ion flow through a membrane nanopore to identify the nucleobases of single-stranded DNA [51]. Alternative isoforms and tumor-specific isoforms resulting from aberrant splicing are often observed during liver carcinogenesis according to long-read SMRT sequencing. Even more intriguingly, it was shown that unannotated variations of ARHGEF2 (v1 and v3) had biological importance in highlighting two important cancer markers [52]. (a) SMRT sequencing technology A zero-mode waveguide (ZMW) is a customized well in which a DNA polymerase–template combination is immobilized. Only the well bottom was illuminated by ZMWs, resulting in a relatively small detection area. As DNA polymerase creates a new complementary strand, nucleotide incorporation causes the fluorescence emission to be monitored. (B) Nanopore sequencing. An electrically resistive membrane contains a protein nanopore, through which double-stranded DNA is unwound and passed. Ionic flow (current) across the membrane is disrupted as the DNA strand passes through it; these variations are monitored to determine the composition of the DNA strand [30] Figure 8.

### Western blotting

**Figure 9.**
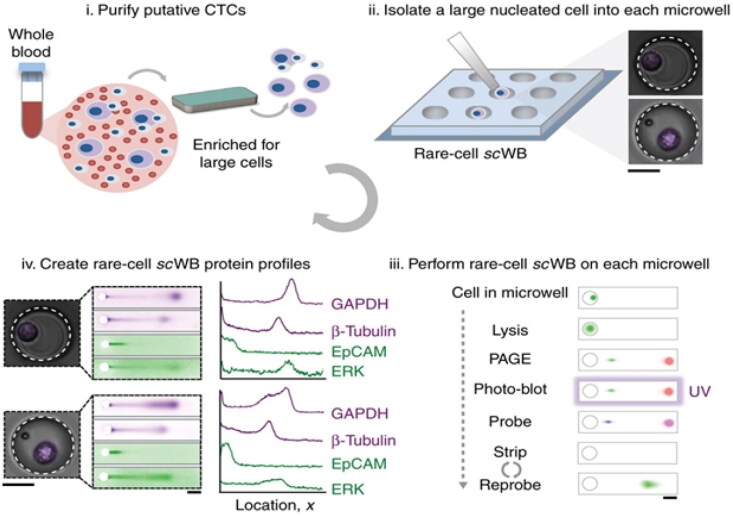
Profiling protein expression using microfluidic western blotting in circulating tumour cells (Adapted from Sinkala E et al.) [54].

In medical and scientific research, western blotting is an effective method widely employed to recognize and isolate various important proteins, especially those that are less common in critical diagnostic samples [53]. Similar to several other illnesses, Ewing sarcoma may be diagnosed using western blotting (Figure 9), which offers insights into the expression of proteins under various cell conditions, relative to other tissues, and in response to molecular alterations [55].

### Fluorescence *in situ* hybridization (FISH)

**Figure 10.**
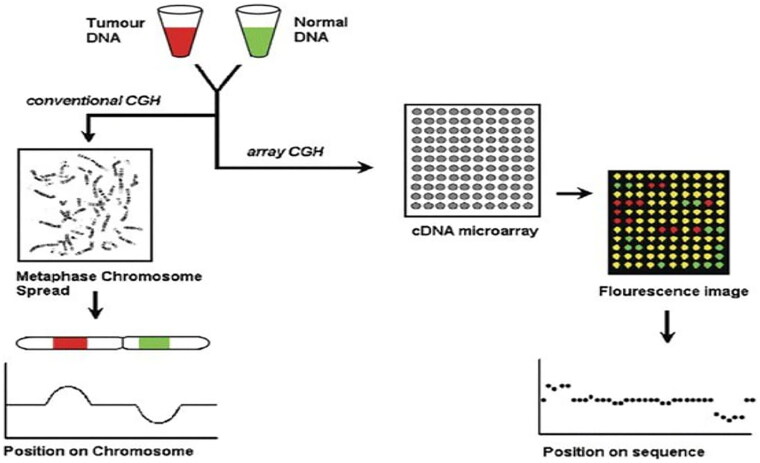
FISH (Adapted from Shakoori AR) [57].

FISH, a technique for identifying macromolecules, is a recent advancement in the scientific field of cytology [56]. FISH analysis has been shown in (Figure 10) and is one of the most effective techniques for noticing individual chromosome numerical aberrations in metaphase, interphase, and paraffin-block nuclei [58]. It is feasible to identify tumor-specific abnormalities using FISH method [59]. One noninvasive supplementary method for identifying bladder cancer is the urine-based FISH test [60]. There is a strong correlation between the number of positive FISH sites and the outcome of the FISH test and tumor recurrence [60]. FISH technology has proven to be highly advantageous in helping physicians to identify and diagnose cervical cancer earlier. This is especially true for women, who are more likely to develop high-grade and low-grade squamous intraepithelial lesions (HSIL and LSIL) [61]. Comparative genome hybridization (CGH), among the most significant developments in the FISH method for genome-wide screening, has been introduced [57].

### Comparative genomic hybridization (CGH)

**Figure 11.**
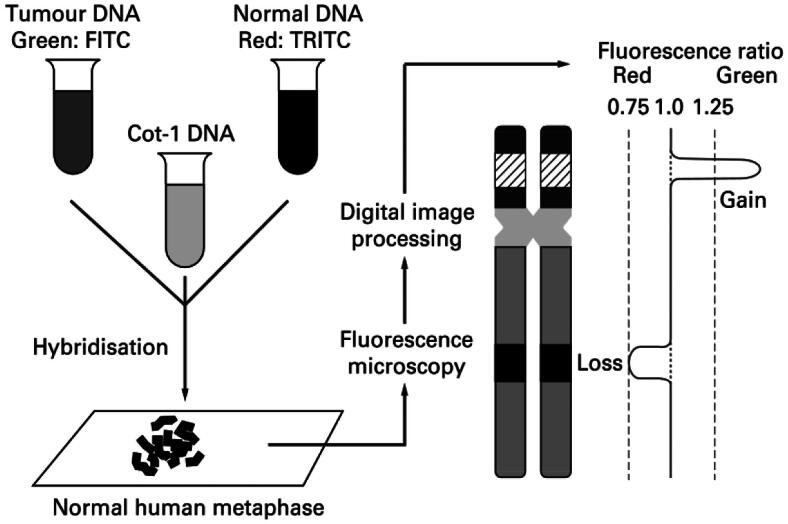
The CGH principle (Adapted from Weiss MM et al.) [64].

**Figure 12.**
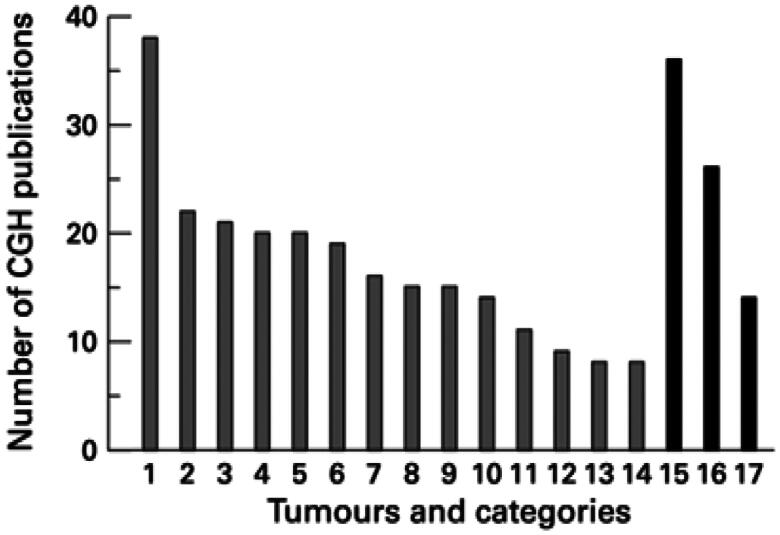
CGH was used to analyze the diversity of tumors and the sorts of reports (1992–1998) (Adapted from Weiss MM et al.) [64].

The CGH approach was developed in 1992 [62] as a genome-wide screening investigation using FISH technology [63]. It is possible to determine differences without requiring cell culture to determine the chromosomal copy number [64]. Schematic representation of the CGH method. Green and red fluorochromes are used to identify the tumor and reference DNA, respectively, and are then hybridized to typical metaphase spreads. For every chromosomal location along the axis of chromosomes, digital quantification of the green-to-red signal ratios was performed using images of the fluorescent signals [64] Figure 11. CGH was used to analyze the diversity of tumors and types of reports (1992–1998). Neurological tumors are the first category, followed by male genital tract tumors, urinary tract tumors, breast cancer, haematological diseases, digestive tract tumors, lung cancer, female genital tract tumors, soft tissue tumors, lymphomas, oral tumors, bone cancer, neuroendocrinal tumors, reports on technology, reviews, and other kinds of reports; skin cancer [64] Figure 12.

### Single nucleotide polymorphism array (SNP)

**Figure 13.**
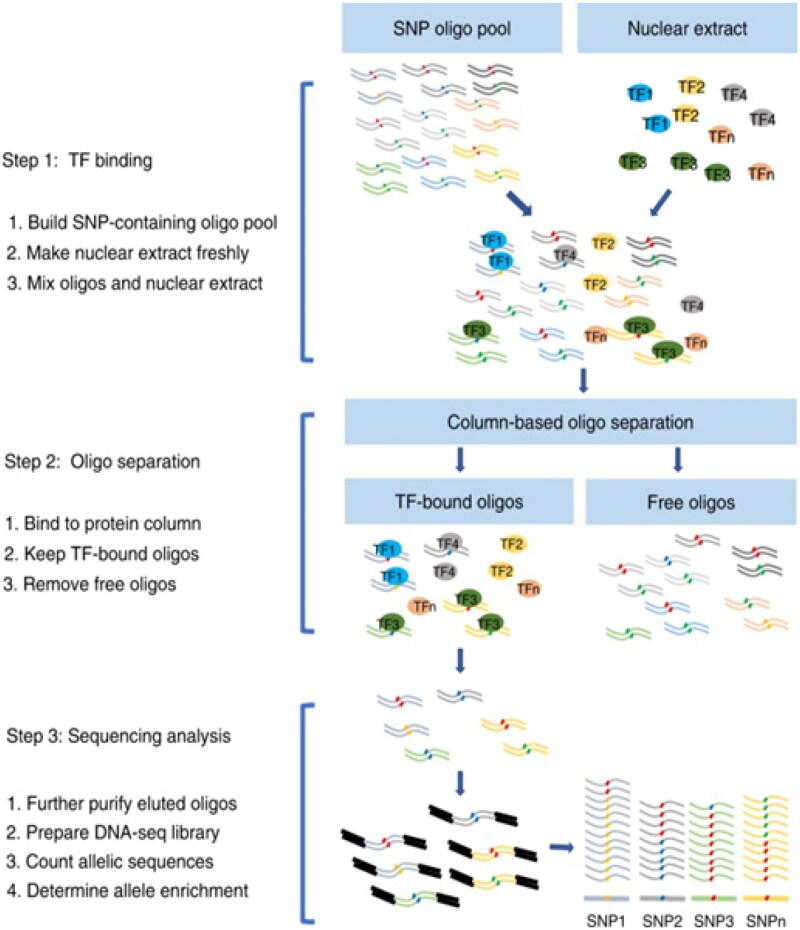
The process of SNPs-seq. (Adapted from Zhang P et al.) [71].

SNP arrays are perfect tools for detecting germline and somatic genetic variations linked to cancer [65]. Chromosomes In cancer genomes, areas of somatic uniparental disomy (UPD) have also been identified by SNP array analysis [66]. SNP array-based genome-wide copy number profiling is becoming more common in the clinical diagnostic workup of melanocytic malignancies [67]. SNP array ­genotyping can identify changes in DNA methylation, genetic copy number, and heterozygosity loss in cancer cells [68]. The origin of ovarian teratomas is revealed by the SNP array [69]. SNP array data from many liver cancer specimen locations have been mapped to show the spatiotemporal ­relationships between immune cells and malignancy [70]. Three crucial stages comprise SNP-seq: nuclear ­protein binding of SNP-containing oligos, separating protein-bound from protein-free oligos, and ­preparing and ­analyzing sequencing libraries [71] Figure 13.

### Proteomic technique

**Figure 14.**
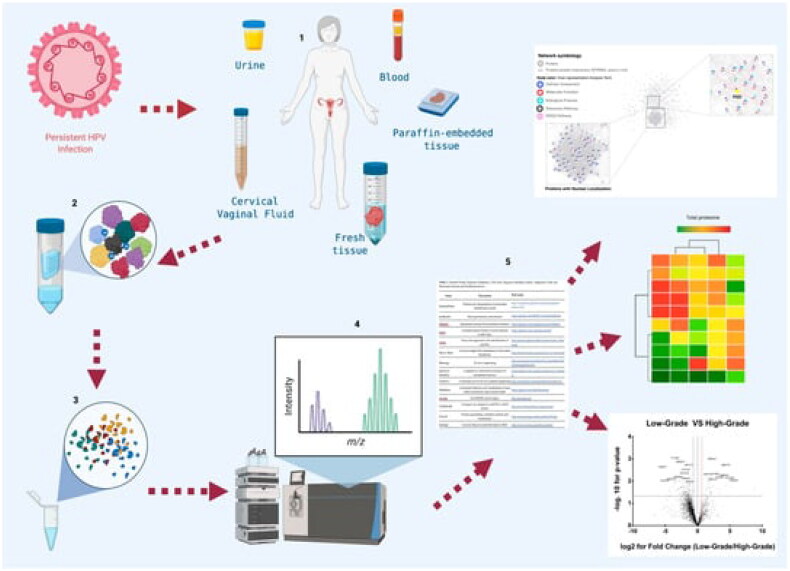
Workflow in proteomic studies in cervical cancer (Adapted from Ding Z et al.) [72].

In the field of cancer diagnostics, proteomic technologies (Figure 14) have become a significant complement to genomic and antibody-based methods [73]. Mass spectrometry, laser capture microdissection, 2-D gel electrophoresis, protein patterns, and identification of molecular markers of cancer are examples of significant technologies [73]. Similar to liquid biopsy, single-cell proteomics has revolutionized all areas of cancer biomarker research [72]. The strategic use of proteomic tools in sarcoma research is growing [74]. To date, proteomic research efforts have focused on characterizing histological subtypes to enhance biological comprehension and find potential diagnostic, prognostic, and predictive biomarkers for clinical application [74].

Proteomics technology can help advance cancer research in the following ways [75]:

- Development of molecular detection (biomarker discovery) of cancer for diagnostic Proposals [76].
- Proteomics enhances our understanding of the molecular pathophysiology (cell signalling) involved in cancer [77].
- Personalized cancer treatment is made possible by drug targeting, which integrates the therapeutic and diagnostic aspects of cancer care [78].
- Improve the cancer categorization [75].

### Immunohistochemistry (IHC)

IHC is an effective tool that seeks to recognize certain antibodies in cells and tissues by utilizing the unique binding that occurs between an antibody and an antigen. Light microscopy is the most frequently used method for identifying and examining these antigens [79]. Compared with other studies in the literature, our health system’s immunohistochemistry screening rates for endometrial cancer in women were lower, and screening results varied according to the patient’s age, ethnicity, and body mass index [80]. It is advised to use automated immunohistochemistry screening instead of requiring a physician’s order, as this is likely to hinder screening uptake [80]. In large-scale clinical studies, IHC-based categorization enables subgrouping of patients with Triple Negative Breast Cancer (TNBC) and assesses the effectiveness of targeted treatments within certain subgroups. This will help make subtype-specific therapy for TNBC patients a reality [81].

### Chromatin immunoprecipitation (ChIP)

A technique referred to as Chromatin immunoprecipitation (ChIP), shown in Figure 16, quantitatively profiles the connections between the proteins of interest and functional chromatin elements [79]. It involves the implementation of antibody-mediated immunoprecipitation, followed by high-throughput or real-time PCR to determine the corresponding DNA fragment sequencing [83]. ChIP is a potent technique for examining the relationship between functional chromatin elements and chromatin-associated proteins *in vivo* is the ChIP [83].

### Circulating tumour DNA analysis (ctDNA)

The DNA fragments released by tumor cells are known as ctDNAs, and they can offer a molecular profile of malignancy [17]. A possible substitute approach is circulating tumor DNA (ctDNA) analysis, which involves direct evaluation of peripheral blood (a ‘liquid biopsy’) for signs of minimal residual illness, which may eventually be the cause of subsequent clinical recurrence [84]. Recurrence-free survival was maintained with less adjuvant chemotherapy among patients with stage II colon cancer treated with ctDNA guidance [85].

The results of this comprehensive review clearly demonstrate the transformative impact of molecular methods in the field of cancer genetics. Table 1 summarizes the findings, limitations, and advantages of several studies utilizing molecular methods in cancer genetics and flow diagram, Figure 18 depicts the representation of molecular methods in cancer diagnostics. This review underscores how these technologies have empowered researchers and clinicians to identify and understand key genetic alterations that drive malignancy, ranging from point mutations to structural variations. Such insights are instrumental in identifying critical oncogenic drivers and potential therapeutic targets, thus paving the way for precision medicine approaches that can significantly improve patient outcomes.

**Table 1.** Advantages, disadvantages and outcomes of molecular techniques in cancer genetics.

Techniques and technical studies included	Advantages	Disadvantages	Outcomes
Polymerase Chain Reaction (PCR) R. Maheaswari et al. [86]	- Sensitive detection of mutations	- Limited to known mutations - Risk of contamination	Widely applied for detecting genetic alterations in cancer
Maxam-Gilbert Sequencing Method S. Dwivedi et al. [87]	- Base-specific sequencing for mutation analysis	- Radioactive chemicals - Labor-intensive	Early identification of cancer-associated genetic mutations
Sanger Sequencing Method M. Rossing et al. [88]	- Accurate sequencing for mutation detection	- Limited throughput - Labor-intensive	Fundamental in discovering genetic mutations linked to cancers
Automated Sequencing Method J. M. Churko at al [89]	- High-throughput mutation screening	- Expensive equipment - Technical complexity	Accelerated identification of mutations in cancer-related genes
Next Generation Sequencing Method L. Ding et al. [90]	- Comprehensive genomic profiling	- Short read lengths - Data analysis challenges	Revolutionized cancer genomics, enabling large-scale studies
Single-Molecule Real-Time Technology L. Ding et al. [90]	- Long read lengths for detailed mutation analysis	- High error rates - Costly consumables	Improved characterization of complex genetic alterations in cancer
Western Blotting W. M. Freeman and S. E. Hemby et al. [91]	- Protein expression analysis for oncogene detection	- Limited quantification - Time-consuming	Contributed to understanding the role of specific proteins in cancer
Fluorescence *in situ* Hybridization Z. A. Ratan et al. [56]	- Visualizes chromosomal abnormalities in cancer	- Limited resolution - Requires expertise	Essential for identifying structural changes in cancer genomes
Comparative Genomic Hybridization N. A. Bergamo et al. [92]	- Detects chromosomal imbalances in cancer	- Low resolution - Limited ability to detect small changes	Revealed genomic instability and copy number variations in cancer
Proteomic Technique K. Chandramouli et al. [93]	- Comprehensive analysis of cancer proteome	- Data interpretation challenges - Technical complexities	Enhanced understanding of protein alterations in cancer progression
Immunohistochemistry L. L. de Matos et al. [94]	- Visualizes specific proteins in cancer tissues	- Subjective analysis - Limited quantitative data	Crucial for identifying protein biomarkers and tumor subtypes
Circulating Tumour DNA Analysis F. Cheng et al. [95]	- Non-invasive detection of circulating cancer DNA	- Low abundance - Specificity challenges	Revolutionized liquid biopsy for monitoring cancer progression

## Discussion

Our knowledge of cancer genetics has been completely transformed by molecular techniques, which have provided insights into the complexities of tumor growth, progression, and response to therapy. To improve patient outcomes and advance our understanding, this discussion concentrates on the fundamentals of molecular methods in cancer genetics.

The discovery of genetic changes that drive the onset and spread of malignancies is central to the field of cancer genetics. Molecular techniques such as next-generation sequencing (NGS) allow for thorough genomic profiling of cancer.

In the age of precision oncology, in which treatment plans are customized based on the unique genetic composition of each patient’s tumor, molecular techniques are essential. Researchers and medical professionals can identify targetable mutations or abnormal signalling pathways using methods such as DNA sequencing.

**Figure 15.**
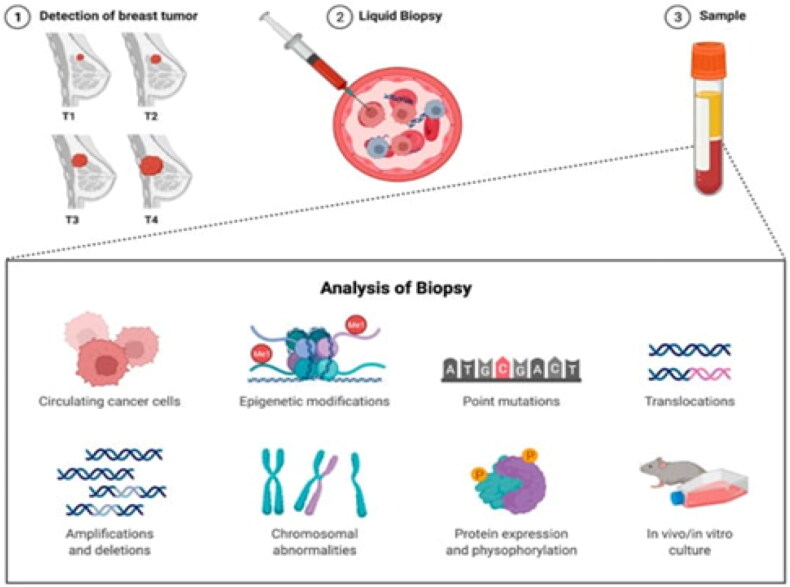
Overview of liquid breast cancer biopsy (Adapted from Hacking SM et al.) [96].

Examining circulating tumor DNA (ctDNA) and other biomarkers in body fluids is referred to as liquid biopsies. Real-time monitoring of tumor dynamics, evaluation of treatment response, and identification of minimal residual disease are all possible using this noninvasive method. Liquid biopsy has become a useful tool in the management of cancer (Figure 15), owing to techniques such as NGS and dPCR, which have increased the sensitivity of the sample.

**Figure 16.**
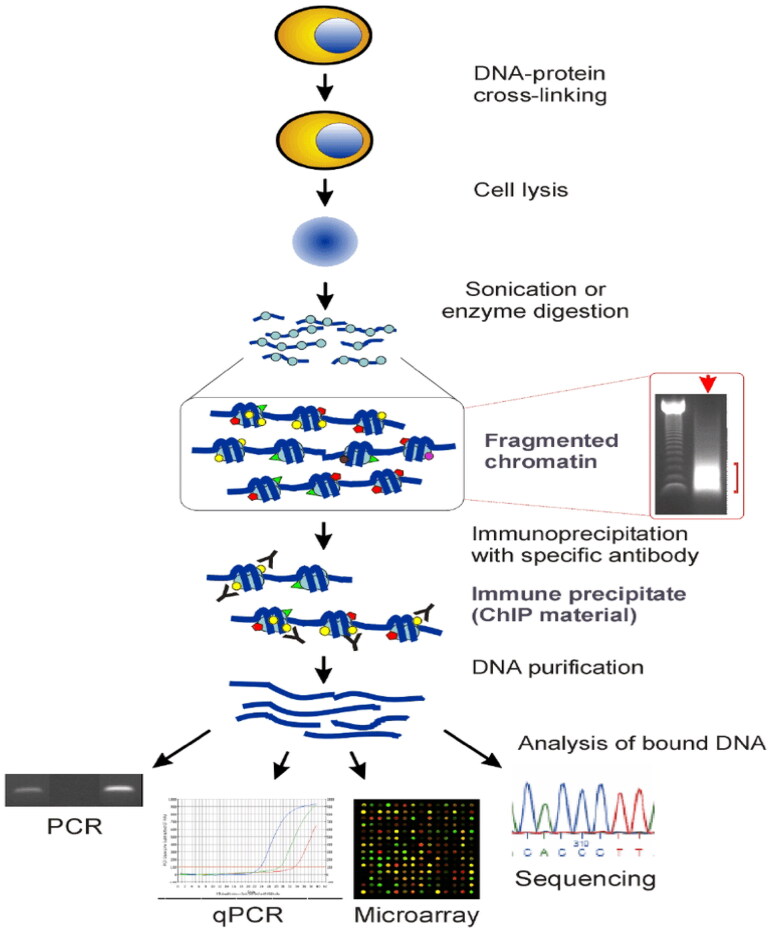
The chromatin immunoprecipitation (ChIP) assay (Adapted from Collas P et al.) [82].

**Figure 17.**
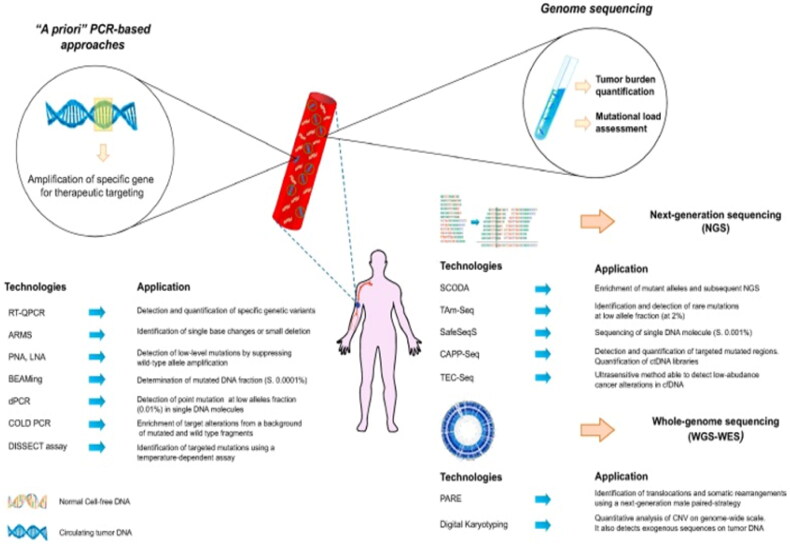
Genome sequencing and ‘a priori’ methods: contrast and use (Adapted from Buono G et al.) [44].

**Figure 18.**
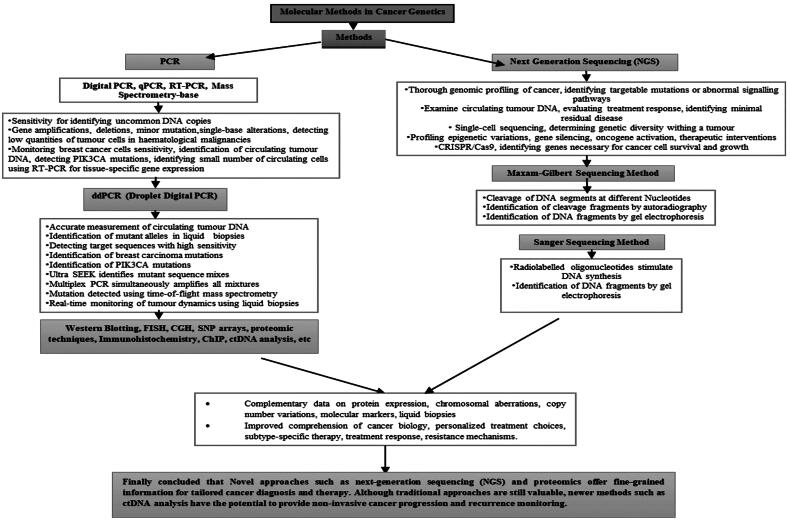
Flow chart representation of molecular methods in cancer diagnostics.

Single-cell sequencing technologies have enabled the dissection of intertumoral heterogeneity through molecular approaches. Researchers can determine genetic diversity within a tumor by examining individual cells. This allows the development of more effective therapeutic approaches that consider the different subclones that contribute to treatment resistance.

Gene expression is modulated by the arrangement of noncoding RNA molecules, histone modifications, and DNA methylation. Through elegant genetic techniques such as ChIP-seq and bisulfite sequencing, researchers can profile these epigenetic variations. Gene silencing, oncogene activation, and therapeutic interventions targeting epigenetic regulators are all possible through an understanding of the epigenetic landscape of cancer.

CRISPR/Cas9 has been used in cancer research to further understand the functional effects associated with specific modifications of the genome. This potent instrument makes it easier to identify the genes necessary for the survival and growth of cancer cells, thereby opening the door for the discovery of new therapeutic targets.

Our understanding of tumor dynamics, treatment response, and resistance mechanisms has been completely transformed by the integration of molecular techniques such as PCR, Maxam-Gilbert sequencing, Sanger sequencing, Next Generation Sequencing (NGS), Single-Molecule Real-Time Technology (SMRT), Western Blotting, Fluorescence *in situ* hybridization (FISH), Comparative Genomic Hybridization (CGH), Single Nucleotide Polymorphism Array (SNP), proteomic ­techniques, Immunohistochemistry (IHC), Chromatin Immunoprecipitation (ChIP), and Circulating Tumour DNA Analysis (ctDNA). These methods provide previously unheard-of levels of sensitivity, precision, and adaptability when identifying genetic and proteomic changes linked to cancer. The development of polymerase chain reaction (PCR) and its derivatives, such as digital PCR (dPCR), real-time quantitative PCR (qPCR), and reverse transcriptase PCR (RT-PCR), has substantially improved our capacity to identify minute mutations, gene amplifications, deletions, and low concentrations of tumor cells. Particularly, real-time PCR and ddPCR allow for the highly sensitive identification of circulating tumor DNA and mutant alleles, even at levels as low as 0.1%. This identification is invaluable in elucidating resistance mechanisms and treatment response.

Furthermore, the development of NGS has revolutionized the field of cancer genomics by making it possible to fully identify all target gene mutations, chromosomal abnormalities, and driver mutations. This has allowed for the development of individualized treatment plans and prognostic evaluations. Our capacity to find alternative isoforms and tumor-specific mutations has increased since the advent of SMRT and nanopore sequencing, opening up new possibilities for targeted treatments.

Complementary data on protein expression, chromosomal aberrations, copy number variations, and molecular markers are provided by Western Blotting, FISH, CGH, SNP arrays, and proteomic techniques. These data improve our comprehension of cancer biology and direct treatment choices. Classifying tumor subtypes and evaluating the effectiveness of treatment are made easier by immunohistochemistry, whereas ChIP analysis provides information on the interactions between chromatin elements and proteins that are involved in the progression of cancer. To further improve treatment outcomes, ctDNA analysis is a promising method for tracking minimal residual disease and forecasting clinical recurrence.

All things considered, the incorporation of these molecular techniques has advanced cancer research and clinical practice, opening the door to more ­efficient prognostic, therapeutic, and diagnostic approaches catered to the specific requirements of each patient.

The significance of ongoing innovations in molecular techniques is highlighted by the dynamic nature of tumors and the emergence of resistance mechanisms.

The future holds great promise for improving our capacity to anticipate treatment responses and discover new therapeutic targets through the integration of artificial intelligence and machine learning algorithms with molecular data. To fully utilize molecular methods to decipher the complexity of cancer genetics and convert these discoveries into tailored and efficient treatments for cancer, collaboration among medical professionals, researchers, and bioinformaticians is crucial.

All things considered, cutting edge technologies like NGS and proteomic techniques offer higher precision, comprehensive insights, and the potential for personalized cancer diagnosis and treatment, even though conventional methods like PCR and sequencing techniques still have their value. Furthermore, there is potential for non-invasive monitoring of cancer progression and recurrence through the use of developing techniques such as ctDNA analysis.

### Limitation

Despite promising advancements in molecular methods for cancer genetics, one significant limitation lies in the accessibility and affordability of these technologies. Although they hold immense potential for improving patient outcomes and guiding personalized treatment strategies, the high costs associated with molecular diagnostics may limit their widespread adoption, particularly in resource-limited settings. This could exacerbate the existing disparities in cancer care, as access to cutting-edge molecular methods may be disproportionately available to certain populations or regions, hindering the realization of their full clinical utility on a global scale. Efforts to address these limitations through cost-reduction strategies, technology transfer initiatives, and equitable distribution policies are essential to ensure that all patients, regardless of socioeconomic status or geographic location, can benefit from advancements in cancer genetics.

Furthermore, many recent advancements in technology concerning cancer genetics were not focused on in this review, despite the publication of many articles. The search may have missed more recent advancements or changes in the field’s landscape of molecular cancer treatments because it does not specify when publications must be included. As a result, it might not have included the most recent developments in the field.

## Conclusion

After conducting an in-depth study of the molecular methods in cancer genetics, it is evident that these cutting-edge technologies have revolutionized the field of oncology, providing researchers and clinicians with powerful tools to unravel the complexities of cancer at the genetic level. The integration of molecular methods has not only enhanced our understanding of cancer etiology, progression, and treatment response but has also opened new avenues for personalized medicine and targeted therapies, leading to improved patient outcomes. Since the search does not indicate when publications must be included, it might have overlooked more recent developments or shifts in the field’s landscape of molecular treatments for cancer. It might not have included the most recent advancements in the field as a result.

### Future prospect

The field of cancer genetics’ molecular techniques holds great promise due to continuous technological advancements and innovations. These methods offer unmatched precision, sensitivity, and adaptability in identifying genetic and proteomic changes associated with cancer. They range from PCR-based approaches like dPCR and qPCR to next-generation sequencing (NGS) and proteomic techniques. Going forward, there is a lot of promise for improving our capacity to anticipate treatment outcomes, identify novel therapeutic targets, and create individualized cancer diagnosis and treatment plans through the integration of artificial intelligence and machine learning algorithms with molecular data. But issues like these technologies’ affordability and accessibility continue to be major roadblocks, especially in environments with limited resources. To guarantee that all patients can profit from the advances in cancer genetics, efforts must be made to address these constraints through cost-reduction techniques and equitable distribution laws. Working together, scientists, physicians, and bioinformaticians will be essential to making the most of molecular techniques in order to unravel the mysteries of cancer genetics and turn these findings into customized, effective cancer treatments.

## Data Availability

Upon a reasonable request, the corresponding author [Dr. Jaishriram Rathored] will provide the data supporting the review’s conclusions.
